# Development of the Chinese coaches’ autonomy-supportive—*laissez-faire* coaching style scale

**DOI:** 10.3389/fpsyg.2024.1412240

**Published:** 2024-07-22

**Authors:** Wen Su, Liqin Wang, Yingmin Ding, Daliang Zhao

**Affiliations:** ^1^Public Courses Teaching Department, Guangzhou Sport University, Guangzhou, China; ^2^Graduate School, Guangzhou Sport University, Guangzhou, China; ^3^College of Leisure and Digital Sports, Guangzhou Sport University, Guangzhou, China

**Keywords:** coaching style, autonomy-supportive coaching style, *laissez-faire* coaching style, coach evaluation scale, coaching style assessment

## Abstract

**Introduction:**

The autonomy-supportive coaching style is recognized for its positive impact on athletes’ well-being and performance. However, the transition of excessive autonomy into a *laissez-faire* coaching style has not been thoroughly examined within the context of coach evaluation scales. Existing scales focus predominantly on the positive dimensions of autonomy support, and do not possess the capabilities to measure outcomes which may be viewed as negative or other outcomes. This study aims to integrate the autonomy-supportive and *laissez-faire* coaching styles within the same measurement framework.

**Methods:**

Our study developed a comprehensive scale to assess both the autonomy-supportive and *laissez-faire* coaching styles, drawing on items from the Sport Climate Questionnaire for autonomy support and adapting items from leadership research for *laissez-faire* coaching. We conducted two studies: the first with 148 athletes to refine the *laissez-faire* items and the second with 460 athletes to validate the full scale, utilizing exploratory factor analysis, confirmatory factor analysis, and correlation analysis. We also measured internal consistency and split-half reliability.

**Results:**

The finalized scale includes a 6-item autonomy-supportive subscale and a 5-item *laissez-faire* subscale. Validation processes confirmed the scale’s construct and criterion validity, alongside its reliability.

**Discussion:**

The Chinese Coaches’ Autonomy-Supportive—*Laissez-Faire* Coaching Style Scale effectively captures both the beneficial and potentially detrimental aspects of coaching styles, addressing a critical gap in the literature and providing a reliable tool for evaluating coaching approaches.

## 1 Introduction

Coach-related factors in the sport environment significantly impact athletes’ growth and development (Côté et al., 2010). Researchers have found improvements in athletes’ subjective vitality (Reinboth and Duda, 2006; Adie et al., 2012), well-being (Haerens et al., 2018), performance (Gillet et al., 2010; Lemelin et al., 2022), mental toughness (Mahoney et al., 2016), and persistence (Pelletier et al., 2001) linked to an autonomy-supportive coaching style. Although an autonomy-supportive coaching style benefits athletes, some coaches express concern that their approach might blur the distinction between providing autonomy support and adopting a *laissez-faire* attitude during practice. This ambiguity arises from the conceptual overlap between the two coaching styles (Stolz and Pill, 2016; SueSee et al., 2021). An autonomy-supportive coaching style includes empathizing with athletes, acknowledging their feelings, and offering opportunities for action and decision-making (Stebbings et al., 2012; Rocchi et al., 2013). Moreover, a crucial skill in this coaching style is to listen carefully to players’ responses, interpreting their significance or completeness (Pill et al., 2021a). By contrast, a *laissez-faire* coaching style is marked by decision-making avoidance, a lack of positive feedback and involvement, and permitting athletes to make their own choices and decisions (Skogstad et al., 2007; Hinkin and Schriesheim, 2008). It is worth noting that, although Self-Teaching Style K is not exactly the same, it shares some similarities. In both styles, the player takes on both the coach and learner roles, making all decisions about the subject matter and activities to achieve their goals (Pill et al., 2021b). However, while both autonomy-supportive and *laissez-faire* coaching styles allow athletes to make choices and decisions, they differ in the level of authorization (Wong and Giessner, 2018). Distinguishing between coaches who adopt an autonomy-supportive or a *laissez-faire* coaching style is crucial for understanding their effectiveness and impact on athlete development.

Despite the potential confusion between the autonomy-supportive and *laissez-faire* coaching styles, existing scales fail to differentiate between the two. The Sport Climate Questionnaire is the most widely used instrument to assess coaching style (Deci and Ryan, 2001); however, this scale measures only the autonomy-supportive dimension and does not include *laissez-faire*. More recently, Delrue et al. (2019) introduced a circumplex model of coaching styles, categorizing four coaching styles into eight more specific approaches: autonomy support (participative and attuning), structure (guiding and clarifying), control (demanding and domineering), and chaos (abandoning and awaiting). This research did not explore the relationship between the autonomy-supportive and *laissez-faire* coaching styles, even though these styles were incorporated into the framework. Additionally, the original *laissez-faire* leadership scale, part of the Multifactor Leadership Questionnaire (Bass and Avolio, 1996), forms the basis for most related scales in leadership styles but only evaluates *laissez-faire* leadership, omitting autonomy-supportive leadership.

This study aims to integrate the autonomy-supportive and *laissez-faire* coaching styles within the same measurement framework. Furthermore, the development of the Chinese Coaches’ Autonomy-Supportive—*Laissez-Faire* Coaching Style Scale will provide coaches with a vital foundation for comprehending the degree of the autonomy-supportive coaching style in their practices.

## 2 Study 1

### 2.1 Methods

#### 2.1.1 Participants

Study 1 encompassed 148 athletes from Guangdong Province, consisting of 77 men and 71 women, with ages spanning 13–30 years (M = 20, SD = 3.079). The distribution of age groups was as follows: 13–15 (*n* = 8), 16–20 (*n* = 87), 21–25 (*n* = 47), and 26–30 years (*n* = 6). Training experience ranged from 0–23 years (M = 6.66, SD = 4.033) and was categorized as 0–5 (*n* = 75), 6–10 (*n* = 49), 11–15 (*n* = 20), 16–20 (*n* = 3), and 21–23 (*n* = 1). Participants were involved in athletics (*n* = 100), martial arts (*n* = 39), and gymnastics (*n* = 9). The Institutional Review Board of Guangzhou Sport University approved all procedures. All participants or their guardians provided written informed consent.

#### 2.1.2 Measures

The *laissez-faire* leadership subscale is adapted from the Multifactor Leadership Questionnaire (Bass and Avolio, 1996). A 7-item preliminary scale was formulated by integrating the *laissez-faire* leadership subscale with the sports environment (Avolio et al., 1999; Skogstad et al., 2007; Hinkin and Schriesheim, 2008; Xirasagar, 2008). A defining characteristic of the *laissez-faire* coaching style is inaction, as reflected in “My coach lets me handle training challenges independently.” Responses were evaluated using a 5-point Likert scale (1 = strongly disagree, 5 = strongly agree).

#### 2.1.3 Data collection and analysis

Data were collected online via the popular Chinese professional survey platform Wenjuanxing.^1^ Following approval from the program center’s head, athletes were given the link to complete the scale. SPSS 25.0 facilitated data analysis, including item-total and item-item correlation analyses for item elimination. Items with correlation coefficients with an overall score below 0.6 were excluded, alongside those with low correlation coefficients with other items.

### 2.2 Results

#### 2.2.1 Item-total and item-item correlation analyses

Initially, the correlation coefficients between each item and the overall score of the *laissez-faire* coaching style subscale were evaluated. It was determined that no items needed to be removed after reviewing items with correlation coefficients less than 0.6. Subsequently, two items were identified for deletion based on the item-item correlation analysis results, specifically, “If I do not seek help from my coach, my coach will not offer assistance” and “My coach lets me handle training challenges independently.” These items’ correlation coefficients with other items consistently fell below 0.6. They were excluded to ensure dimensional consistency and relevance to the context of Chinese competitive sports.

## 3 Study 2

### 3.1 Methods

#### 3.1.1 Participants

Study 2 involved 460 athletes from Guangdong Province, consisting of 239 men and 221 women, with ages ranging from 10–32 years (M = 18.78, SD = 3.763). The age groups were as follows: 10–15 (*n* = 90), 16–20 (*n* = 237), 21–25 (*n* = 111), 26–30 (*n* = 21), and 31–32 years (*n* = 1). Training experience ranged from 0–23 years (M = 8.49, SD = 3.910) and was categorized as follows: 0–5 (*n* = 118), 6–10 (*n* = 223), 11–15 (*n* = 97), 16–20 (*n* = 20), 21–23 (*n* = 2). Participants engaged in various sports including fencing (*n* = 58), volleyball (*n* = 49), gymnastics (*n* = 45), badminton (*n* = 42), weightlifting (*n* = 29), swimming (*n* = 28), basketball (*n* = 28), table tennis (*n* = 28), sanda (*n* = 27), diving (*n* = 25), athletics (*n* = 23), artistic swimming (*n* = 20), trampolining (*n* = 20), Wushu (*n* = 19), water polo (*n* = 16), and tennis (*n* = 3). All procedures were approved by the Institutional Review Board of Guangzhou Sport University. All participants or their guardians provided written informed consent.

#### 3.1.2 Measures

The autonomy-supportive subscale, initially derived from the health domain (Williams and Deci, 1996) was later adapted for the sports context. This scale measures athletes’ perceptions of autonomy support from their coaches, exemplified by “I feel that my coach provides me with choices and options.” The adjusted scale showed robust psychometric properties (Reinboth et al., 2004). A 7-point Likert scale was used to rate responses (1 = strongly disagree, 7 = strongly agree).

Study 2 implemented the Self-Esteem Scale and the Subjective Vitality Scale to validate the study’s scale. The Self-Esteem Scale is known for its reliability in assessing positive and negative self-perception, contributing to overall self-worth (Rosenberg, 1965; Wang et al., 1999), including items like “I feel that I have a number of good qualities,” rated on a 4-point Likert scale (1 = strongly agree, 4 = strongly disagree). The Subjective Vitality Scale measures participants’ positive vitality and energy (Ryan and Frederick, 1997), with items such as “I feel alive and vital right now.” The Chinese version of the Subjective Vitality Scale demonstrated high internal consistency (CR = 0.87) in sports settings (Liu and Chung, 2014), with responses rated on a 7-point Likert scale (1 = strongly disagree, 7 = strongly agree).

#### 3.1.3 Data collection and analysis

Data were collected online via the popular Chinese professional survey platform Wenjuanxing.^2^ Following the program center head’s approval, athletes were sent the link to complete the scale. For analysis, Study 2 used SPSS 25.0 and AMOS 28.0. The exploratory factor analysis removed items with factor loadings below 0.5 or with significant cross-loadings. The confirmatory factor analysis evaluated construct validity. Excellent-fit indices included CFI ≥ 0.95, TLI ≥ 0.95, and RMSEA ≤ 0.06 (Hu and Bentler, 1999), with acceptable-fit indices of CFI ≥ 0.90, TLI ≥ 0.90, RMSEA ≤ 0.08 (Browne and Cudeck, 1992; Hu and Bentler, 1999), and PNFI ≥ 0.60 (Netemeyer et al., 1990). The appropriate range of *χ*^2^/df was recommended to be between 2 and 5 (Schumacker and Lomax, 2004; Côté et al., 2017). Furthermore, correlation analysis was performed for reliability and validity tests.

### 3.2 Results

#### 3.2.1 Exploratory factor analysis

The exploratory factor analysis on data comprising 11 items related to the autonomy-supportive and *laissez-faire* coaching styles used principal component analysis with two factors for extraction and employed varimax rotation. The sample suitability test (Kaiser-Meyer-Olkin = 0.92) and spherical test (*χ*^2^ = 3923.27, *p* < 0.001) verified the sample’s appropriateness for factor analysis. The autonomy-supportive subscale’s factor loadings ranged from 0.76 to 0.88, while the *laissez-faire* subscale’s loadings varied from 0.74 to 0.88, with no items displaying significant cross-factor loadings (Table 1). Each subscale had an eigenvalue greater than 1, cumulatively contributing 74.81% to the variance. The autonomy-supportive dimension’s eigenvalue was 4.47, explaining 40.61% of the interpretable variance, and the *laissez-faire* dimension’s eigenvalue was 3.76, accounting for 34.20%.

**Table 1:** Exploratory factor analysis: descriptive statistics and factor loadings.

Item	M	SD	Autonomy-supportive	*Laissez-faire*
Autonomy-supportive coaching style				
I feel that my coach provides me with choices and options	5.10	1.29	**0.76**	
I feel understood by my coach	4.72	1.49	**0.85**	
My coach conveyed confidence in my ability to do well in athletics	5.04	1.34	**0.84**	
My coach encouraged me to ask questions	5.13	1.34	**0.82**	
My coach listens to me regarding how I would like to do things	4.86	1.48	**0.88**	
My coach tries to understand my perspective before suggesting a new way of doing things	4.60	1.52	**0.86**	
*Laissez-faire* coaching style				
My coach remained inactive despite being aware of my consistently declining performance	2.72	1.38		**0.83**
My coach fails to provide feedback on whether my performance is satisfactory	2.69	1.42		**0.88**
Unless the situation is dire, my coach does not communicate with me	2.66	1.41		**0.84**
Once the training plan is established, my coach shows no interest in my training progress	2.51	1.34		**0.85**
Whenever I require assistance, my coach is invariably unavailable	2.72	1.38		**0.74**

Items of the autonomy-supportive coaching style were derived from the Sport Climate Questionnaire (https://selfdeterminationtheory.org/sport-climate-questionnaire/). Items of the laissez-faire coaching style were modified from laissez-faire leadership concepts (Bass and Avolio, 1996). The primary factor loadings are in bold. M, mean; SD, standard deviation.

#### 3.2.2 Confirmatory factor analysis

The confirmatory factor analysis was conducted with two dimensions (autonomy-supportive and *laissez-faire* coaching styles) serving as potential variables. The analysis yielded an acceptable fit to the data: *χ*^2^/df = 4.14, RMSEA = 0.08, CFI = 0.97, TLI = 0.96, PNFI = 0.75.

To determine if the autonomy-supportive and *laissez-faire* coaching styles could be conceptualized as a single dimension, all items were used as observed variables, while a single latent variable was utilized to model the structural equations. The results demonstrated the following fit indices: *χ*^2^/df = 27.67, RMSEA = 0.24, CFI = 0.70, TLI = 0.63, PNFI = 0.55. The model did not meet acceptable standards, indicating that the autonomy-supportive and *laissez-faire* coaching styles represent two distinct dimensions rather than a single dimension (Table 2).

**Table 2:** Two-dimension and single-dimension fitting indicators.

	χ^2^/df	RMSEA	CFI	TLI	PNFI
Two-dimensional model	4.14	0.08	0.97	0.96	0.75
One-dimensional model	27.67	0.24	0.70	0.63	0.55

#### 3.2.3 Correlation analysis between the autonomy-supportive and *laissez-faire* coaching styles

A significant negative correlation was found between the two subscales (autonomy-supportive and *laissez-faire* coaching styles) (Table 3).

**Table 3:** Correlation analysis results between autonomy-supportive and *laissez-faire* coaching dimensions.

	Autonomy-supportive	*Laissez-faire*
Autonomy-supportive	1	
*Laissez-faire*	−0.50^**^	1

^**^p < 0.01.

#### 3.2.4 Reliability analysis

Reliability tests were conducted to assess the consistency of the autonomy-supportive and *laissez-faire* coaching styles (Table 4). The findings indicated that the internal consistency for both dimensions was excellent. Furthermore, the split-half reliability for both styles achieved high reliability indices.

**Table 4:** Reliability measures of the Chinese Coaches’ Autonomy-Supportive—*Laissez-Faire* Coaching Style Scale.

	Internal consistency reliability	Split-half reliability
Autonomy-supportive	0.93	0.90
*Laissez-faire*	0.91	0.84

#### 3.2.5 Correlation analysis between the subscales and two additional scales

A correlation analysis was performed to evaluate the validity of the scale, incorporating two additional factors: self-esteem and subjective vitality (Table 5). The analysis revealed that self-esteem had a significant positive relationship with the autonomy-supportive coaching style and a substantial negative relationship with the *laissez-faire* coaching style. Likewise, subjective vitality showed a significant positive correlation with the autonomy-supportive coaching style and a notable negative relationship with the *laissez-faire* coaching style.

**Table 5:** Correlation analysis results between coaching styles and psychological factors: self-esteem and subjective vitality.

	Autonomy-supportive	*Laissez-faire*
Self-esteem	0.37^**^	−0.22^**^
Subjective vitality	0.58^**^	−0.22^**^

^**^p < 0.01.

## 4 Discussion

The purpose of this study was to develop a scale to assess Chinese coaches’ autonomy-supportive and *laissez-faire* coaching styles, providing a comprehensive foundation for evaluating coaching behavior. This scale included 11 items, with six items dedicated to measuring the autonomy-supportive coaching style and five items for assessing the *laissez-faire* coaching style. Evidence from this study supports the reliability and validity of the scale.

The autonomy-supportive subscale utilized the well-known Sport Climate Questionnaire, whereas the *laissez-faire* subscale, derived from the Multifactor Leadership Questionnaire, was adjusted to suit the sports context. After removing specific *laissez-faire* items, these two subscales formed a unique scale structure with contrasting directions. Unlike other instruments that assess coaching behavior, such as the Interpersonal Behaviors Questionnaire and the Coaches’ Interpersonal Style Questionnaire, which measure dimensions like coaches’ autonomy support and autonomy thwarting simultaneously (Rocchi et al., 2017; Pulido et al., 2018), our scale recognizes these dimensions as inherently opposing and interconnected, underscoring the complexity of coaching styles. It acknowledges that a coach can adopt any coaching styles, as coaching styles are choices rather than personal characteristics. Knowledge of decision making between athlete and coach allows for the selection of different styles and encourages a “non-versus” approach to coaching, where different styles are not seen as better or worse, but as mean to achieve different outcomes (Mosston and Ashworth, 2008). This approach frames coaching as a structural act of teaching rather than one driven by personal preference (Pill et al., 2021c).

The notion of autonomy support is derived from self-determination theory within the realm of coaching styles, while *laissez-faire* originates from managerial leadership styles. To validate this scale, the study introduced two additional variables: self-esteem and subjective vitality. Aligning with previous research (Coatsworth and Conroy, 2009; Balaguer et al., 2012; Cheval et al., 2017), our findings indicate a positive association between the autonomy-supportive coaching style and both self-esteem and subjective vitality. Existing theories suggest that athletes’ basic psychological needs may mediate the impact of coaching style on self-esteem (Coatsworth and Conroy, 2009; Cheval et al., 2017). Moreover, athletes’ perceptions of autonomy-supportive coaching positively predict psychological need satisfaction, which, in turn, positively affects subjective vitality (Balaguer et al., 2012). Conversely, our study reveals a negative correlation between the *laissez-faire* coaching style and both self-esteem and subjective vitality, consistent with research indicating a negative association between *laissez-faire* coaching and positive psychological outcomes (Skogstad et al., 2007; Robert and Vandenberghe, 2022). Specifically, *laissez-faire* leadership has been linked to increased depressive symptoms and reduced positive mental health (Robert and Vandenberghe, 2022), possibly due to diminished autonomy and role clarity, leading to decreased well-being (Lundmark et al., 2022; Desgourdes et al., 2024). Our study also finds that the *laissez-faire* coaching style has a negative effect on positive psychological factors including self-esteem and subjective vitality. By bridging the gap between coaching and leadership styles, this study developed a scale that encompasses various coaching styles and offers valuable insights for future coaching practices.

The development of the Chinese Coaches’ Autonomy-Supportive—*Laissez-Faire* Coaching Style Scale integrates coaching and leadership styles, supported by statistical evidence of its reliability and validity. Nevertheless, the study has limitations. First, it primarily surveyed athletes from provincial sports teams, which may limit the generalizability of the findings to different levels of sports teams. Future research should assess the scale’s applicability to diverse athlete groups. Second, while this scale focuses on the “degree” of autonomy support from coaches, similar considerations may apply to other coaching styles, such as controlling styles. Third, although the scale incorporated insights from leadership research, further empirical studies are needed to elucidate its relationship with other psychological factors.

## Data availability statement

The original contributions presented in the study are publicly available. The data can be found here: https://osf.io/5h6te/.

## Ethics statement

The studies involving humans were approved by the Institutional Review Board of Guangzhou Sport University. The studies were conducted in accordance with the local legislation and institutional requirements. Written informed consent for participation in this study was provided by the participants’ legal guardians/next of kin.

## Author contributions

WS: Methodology, Writing – original draft, Writing – review & editing. LW: Methodology, Writing – original draft. YD: Methodology, Writing – original draft. DZ: Conceptualization, Funding acquisition, Resources, Supervision, Writing – review & editing.

## Footnotes

^1^ https://www.wjx.cn/

^2^ https://www.wjx.cn/
